# Clinical impact of irreversible electroporation ablation for unresectable hilar cholangiocarcinoma

**DOI:** 10.1038/s41598-020-67772-2

**Published:** 2020-07-02

**Authors:** Chih-Yang Hsiao, Po-Chih Yang, Xiaoyong Li, Kai-Wen Huang

**Affiliations:** 1 Graduate Institute of Clinical Medicine, National Taiwan University College of Medicine, Taipei City, 10002 Taiwan; 2 Department of Surgery, National Taiwan University Hospital, 7 Chung-Shan South Rd, Taipei, 10002 Taiwan, ROC; 3 Department of Traumatology, National Taiwan University Hospital, Taipei City, 10002 Taiwan; 4 Center for Organ Transplantation and Liver Disease Treatment, Fu Jen Catholic University Hospital, New Taipei City, 24352 Taiwan; 5 Department of Hepatopancreatobiliary Surgery, The Fifth Affiliated Hospital, Zhengzhou University, Zhengzhou, 450052 China; 6 Hepatitis Research Center, National Taiwan University Hospital, Taipei City, 10002 Taiwan

**Keywords:** Cancer therapy, Bile duct cancer

## Abstract

Irreversible electroporation (IRE) is a non-thermal ablation modality that has been shown to be safe and effective in its application to tumors that are close to risky areas. This study aims to assess the safety and efficacy of IRE for unresectable hilar cholangiocarcinoma. Nine patients from two medical centers in Asia received IRE treatment between June 2015 and July 2017. Before IRE treatment, percutaneous biliary decompressions had been performed on eight patients, and internal stenting had been performed on one patient. All patients tolerated the procedure well without high-grade complications. The ablated tumors had constant size without contrast enhancement for more than three months in eight patients and the level of CA19-9 decreased significantly in all patients. The percutaneous biliary drainage tube was removed from two patients with recanalization of the bile duct. The internal stent in one patient was removed without further stenting. The median overall survival period was 26 months, and the progression-free survival was 18 months. Bile ducts remained narrow in the majority (2/3) of the treated patients. Nevertheless, IRE ablation of unresectable hilar cholangiocarcinoma involving vital structures is a safe and feasible primary treatment for local tumor control and is effective in prolonging survival.

## Introduction

Hilar cholangiocarcinoma, also known as a Klatskin tumor, is a form of ductal adenocarcinoma involving a common bile duct and the bifurcation of intrahepatic ducts. It is the most common type of bile duct cancer, accounting for approximately 60–70% of total cholangiocarcinomas^1^. The Bismuth-Corlette staging system classifies hilar tumors by the extension and location of hepatic ductal infiltration. Unfortunately, the majority of patients have an advanced disease that precludes radical resection due to either local vascular invasion, hepatic extension, or distant metastasis, in which resectability is less than 30%^2^.

There are no effective modalities for patients who are not suitable for surgical resection. Empirical radiotherapy and chemotherapy with gemcitabine-based regimens can achieve a response rate of only 7–27%, and it cannot sufficiently prolong a patient’s lifespan. Patients usually succumb to obstructive jaundice and related complications. Long-term patency of biliary drainage is the only solution proven to be helpful for patient survival^3^, but even with successful biliary stenting, the average survival period is only 6–8 months^4^.

Irreversible electroporation (IRE) is a new non-thermal ablative modality that induces cancer cell apoptosis in tumors through the destruction of cell membranes by electroporation^5^. Recently, the technique has been routinely applied for the treatment of pancreatic cancer, liver cancer, and prostate cancer^6,7^, and its safety and efficacy have been documented. However, there remains a dearth of literature on the role of IRE in hilar cholangiocarcinoma.

In this study, we report our experience with IRE treatment in nine patients with unresectable hilar cholangiocarcinoma from two medical centers in Asia. These patients had been treated with only palliative percutaneous transhepatic cholangiography and drainage (PTCD) before IRE treatment. The aim of the study was to investigate pilot results and explore the clinical efficacy and safety of IRE treatment for hilar cholangiocarcinomas.

## Materials and methods

### Patients

This retrospective study was approved by the ethics committee and the institute review board of National Taiwan University Hospital (Protocol ID: 201210007DIC), and all study methods were performed in accordance with the relevant guidelines and regulations. All study subjects provided informed consent prior to participation in the investigation. The study retrospectively included nine patients with unresectable hilar cholangiocarcinoma from National Taiwan University Hospital and the Fifth Affiliated Hospital of Zhengzhou University who were seen from June 2015 to July 2017, and the data were collected in December 2019.

The demographic data and the patient characteristics are shown in Table 1. Four patients were male, and five patients were female. The mean age was 63.6 ± 9.03 (range 41–83) years. The clinical presentations before diagnosis included jaundice, pruritus, and clay-colored stool. Preoperative diagnoses were made by contrast-enhanced computed tomography (CT), contrast-enhanced magnetic resonance (MR) imaging, or positron emission tomography (PET) scans if the image revealed an enhanced soft tissue density within the bifurcation of the common hepatic duct with intrahepatic bile duct dilation. All patients had unresectable hilar cholangiocarcinoma, which was confirmed by intraoperative exploration and tumor biopsies. Seven of the patients had locally advanced disease, and two patients had systemic disease with distant metastasis.

**Table 1:** Baseline characteristics of patients treated with IRE.

Variable	IRE (N = 9)
Age (y), mean ± SD	63.6 ± 9.03
Gender (male/female), n	4/5
BMI (kg/m^2^), mean ± SD	23.6 ± 3.62
Albumin (g/dL), mean ± SD	4.3 ± 0.29
PTCD	8
INR	1.0 ± 0.02
CA19-9 (U/mL), mean ± SD	98.5 ± 62.39
CEA (U/mL), mean ± SD	12 ± 2.39
AST (IU/L), mean ± SD	51.2 ± 53.61
ALT (IU/L), mean ± SD	48.1 ± 41.74
Total bilirubin (mg/dL), mean ± SD	2.9 ± 1.4
Bismuth type, n (%)	
Type III	3
Type IV	6

According to the Bismuth–Corlette classification, three patients had type III disease, six patients had type IV disease, and the mean primary tumor size was 2.8 ± 1.2 cm. The definition of unresectable tumor refers to AJCC (8th edition) stage IV disease (i.e., with any regional lymph node metastasis) or stage III disease (i.e., tumor perforating the visceral peritoneum or involving local extrahepatic structures by direct invasion) without adequate liver reserve to undergo hepatic resection. As for the gross morphology of the tumors, five patients had the periductal or intraductal type, and four patients had the mass forming type based on image studies. All patients had no severe systemic diseases.

All patients had obstructive jaundice at the time of diagnosis. According to imaging examinations, eight patients had complete obstruction of at least one major hepatic duct, and these eight patients had undergone PTCD without an attempt at endoscopic retrograde biliary drainage before IRE treatment. One patient with partial biliary obstruction had undergone endoscopic retrograde biliary drainage to place a plastic stent in a bile duct before IRE treatment.

### Treatment procedure

Before IRE treatment, all patients had undergone routine preoperative evaluations, including complete blood count, liver function, CA-19-9 levels, creatinine kinase levels, coagulation function tests, and electrocardiograms to assess their suitability for IRE treatment. Tumor ablation with IRE was performed under general anesthesia with full paralysis using the Nanoknife System (Angiodynamic, USA) sonography guidance. Five patients underwent IRE by laparotomy, and four patients underwent IRE percutaneously. Patients who underwent IRE by laparotomy also underwent cholecystectomy and regional lymph node dissection.

The ablation zone was planned to cover the whole targeted area including the suspicious tumor area based on imaging, bile ducts that were abnormal or narrowed, and regionally enlarged lymph nodes. Electrodes were inserted under sonography guidance based on the principle of the caudal-cranial direction and parallel to the hepatoduodenal ligament (during laparotomy) or an anteroposterior approach (during percutaneous approach) to avoid injury of the vessels. The ablation area was covered by the electric field created using 3 to 4 unipolar electrodes with an active tip of 1.5–2.0 cm, and the distance between each electrode was 1.5–2.5 cm.

In total, 90–180 electric pulses with a voltage of 2,400–3,000 V/cm were administered between each pair of electrodes, and the pulse length was set at 70–90 ms. After removal of the electrodes, the operator checked whether there was any bleeding or bile leakage, and compression or suturing was applied if it was deemed necessary. An intraperitoneal drain was placed for postoperative monitoring in patients who had undergone laparotomies.

### Post-treatment examination and management

After the IRE procedure, patients were closely monitored in the ward. Empirical antibiotics and intravenous fluid were given with fasting within 24 h. Regular monitoring of complete blood cell count, serum biochemistry profile including muscle enzymes, and the liver and renal functional profiles were determined on postoperative days 1, 3, and 7. Laboratory examinations and contrast-enhanced CT or MR were performed one month after the procedure and then once every three months. A cholangiogram was obtained on postoperative day 7 via PTCD or endoscopic retrograde cholangiopancreatography. No patient underwent postoperative radiotherapy, but two patients underwent postoperative adjuvant chemotherapy.

The efficacy of treatment was assessed by contrast-enhanced CT or MR imaging at one month and three months after the procedure. The treatment was considered effective and complete when the following findings were observed: (1) absence of contrast enhancement of the tumor in the one-month CT/MR image and (2) progressive shrinkage of the tumor in the three-month CT/MR image. The treatment was considered partially effective or not effective when contrast enhancement of the tumor was still present in the one-month CT/MR image or new solid enhanced nodules or enlargement of the tumor mass was detected in the three-month CT/MR image^8^. The percutaneous biliary drainage tube was removed after the procedure within three months if the bile duct was patent without evidence of obstructive jaundice, and it was replaced with an expandable metallic stent if necessary.

### Statistical analysis

Data are expressed as the mean ± standard deviation, and paired t-tests were used to compare the clinical and biochemical data of patients before and after the procedure. A value of *P* < 0.05 was considered statistically significant. Statistical analyses were performed using SPSS 17.0 (SPSS Inc., Chicago, IL).

## Results

### Treatment course

All patients underwent the IRE procedure without complications or severe arrhythmia. Hypertension was noted in some patients, but systolic pressure never exceeded 200 mmHg in any patient. During the procedure, the delivered current was distinctly elevated with a difference of 10–15 A, which shows the effectiveness of electroporation. The lesions in all patients were proven to be adenocarcinoma by intra-operative biopsies. Two of the five patients who underwent lymph node dissection during laparotomy had lymph node metastasis. No patients had high-grade complications according to the Clavien–Dindo classification. No patients had postoperative bleeding, bile leakage, or infection. All patients were discharged within seven days after operation.

Two patients were within the normal range for the tumor marker CA19-9 before and after treatments. The other seven patients had elevated CA19-9 preoperatively, which gradually returned to the normal range by one month after the IRE procedure. The preoperative mean total bilirubin level was 2.9 ± 1.1 mg/dL, and the postoperative mean total bilirubin level one month later was 1.2 ± 0.4 mg/dL. Three months after the operation, trans-catheter cholangiography was performed, and three patients had patent bile ducts. Therefore, their external PTCD tube (n = 2) or plastic internal stent (n = 1) was removed, and one patient with PTCD received internal metallic stent replacement before the removal of the PTCD.

**Figure 1:**
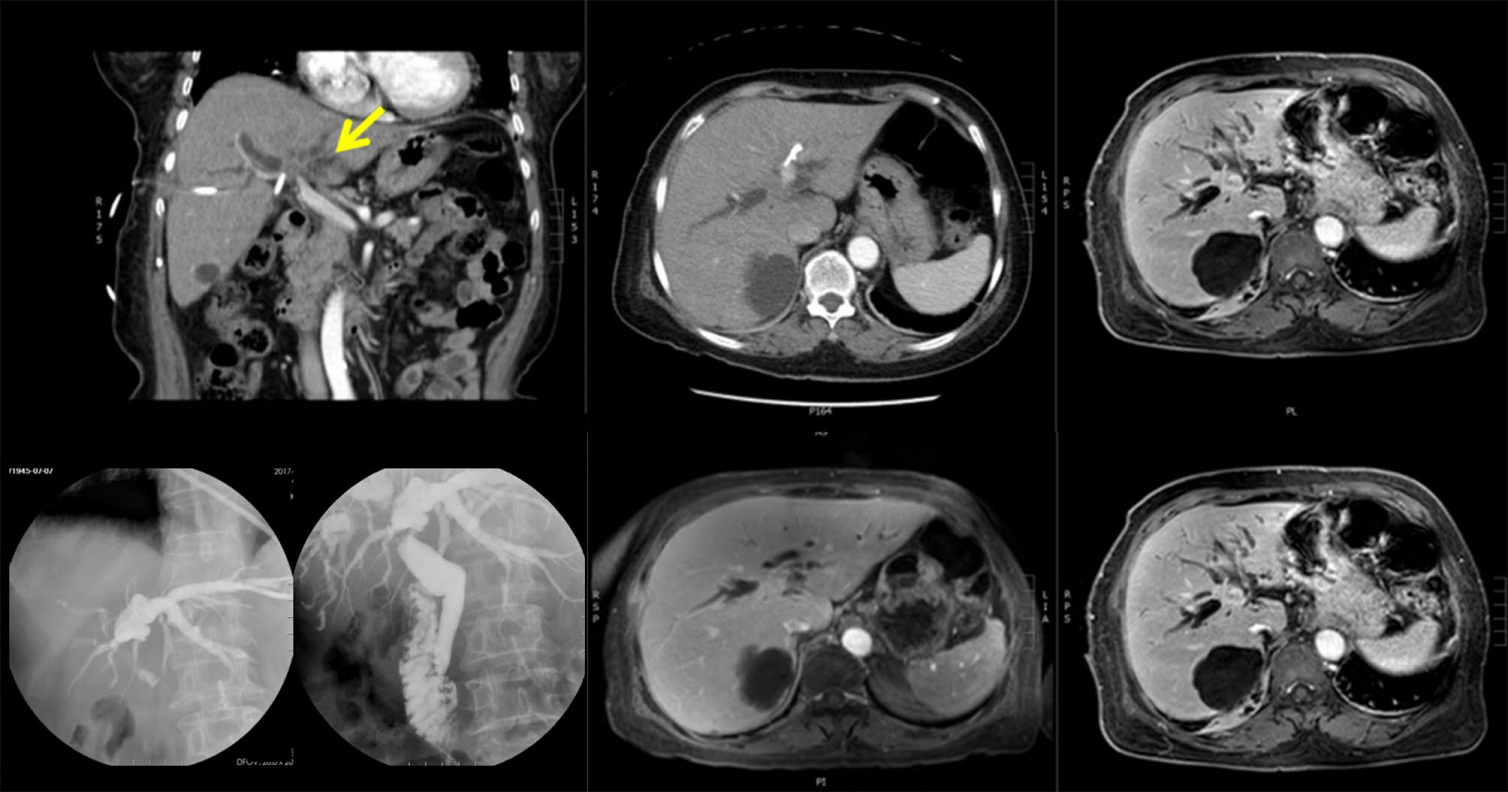
An index patient with unresectable hilar cholangiocarcinoma who underwent IRE treatment. Pre-operative contrast-enhanced abdominal CT/MR image showed hilar tumor with dilated IHD (upper part), whereas post-IRE treatment at 3 months (middle lower part) and 28 months (right lower part) showed good local control (progressive shrinkage of tumor without any contrast-enhancing lesion) with complete response. Trans-catheter cholangiography by injecting contrast medium through the PTCD tube also showed satisfactory results (left lower part). Before IRE treatment, an obstructive bile duct was observed (left), but three months post-IRE treatment, a patent bile duct the (right).

PTCDs were not removed from the other six patients because they still had apparent narrowing of the bile ducts, which was revealed by cholangiogram. Follow-up contrast-enhanced CT/MR at one month after the procedure revealed complete response in eight of the nine patients (Fig. 1). Only one patient had residual enhanced nodules with an enlarged disease area, which suggested a remaining tumor. The same results were observed by CT/MR at three months after IRE, and a complete ablation rate of 88.9% was achieved.

### Follow-up and outcome

All patients survived for at least 12 months after IRE treatment. During follow-up in the first year, three patients had disease progression, including three patients who had regional lymphadenopathy that were suspicious for metastasis. One patient had intrahepatic distant liver metastasis, and two patients had local recurrence in the ablated area, which presented as hyperbilirubinemia and biliary infection (Table 2). Two patients underwent postoperative adjuvant chemotherapy with a gemcitabine-based regimen, and seven other patients refused chemotherapy for personal reasons based on quality of life and health care support.

**Table 2:** Outcome of patients at 12 months after IRE treatment.

Outcome variable	Number (total N = 9)
Survival	9 (100%)
Disease progression	3 (33.3%)
Regional lymphadenopathy	3 (33.3%)
Distant metastasis	1 (11.1%)
Local recurrence	2 (22.2%)
Patent bile duct without internal stent or PTCD	2 (22.2%)
Patent bile duct with internal stent only	1 (11.1%)
Obstructive bile duct with PTCD	6 (66.7%)

Among the nine patients, the follow-up period ranged from 13 to 39 months. The median overall survival time was 26 months (interquartile range 23–32 months), and the median progression-free survival time was 18 months (interquartile range 12–21 months). Seven patients had disease progression during the follow-up period, and four of them died in the 13th, 23rd, 25th, or 32nd postoperative month due to liver failure related to multiple liver metastases and severe infection. Five patients were alive (median OS, 27 months) as of the last follow-up at data collection (aside from one patient who survived 17 months and was lost to follow-up). Two patients were still in a progression-free state at the end of follow-up in the 33rd and 39th months after treatment.

## Discussion

Hilar cholangiocarcinoma has a unique anatomical location with diffuse infiltration along the biliary tree. Resectability is rare, and the prognosis is usually poor. In recent years, the detection of hilar cholangiocarcinoma and thus its incidence have increased because of advances in image modalities and the prevalence of health examinations^9^.

Current treatment options for unresectable hilar cholangiocarcinoma include chemotherapy, radiotherapy, palliative biliary drainage (by percutaneous external drainage or internal drainage with stent placement), radiofrequency ablation, and photodynamic therapy. Chemotherapy and radiotherapy have poor responses^10–12^, and palliative biliary drainage relieves only obstructive jaundice without treating the primary tumor, resulting in a poor survival rate in most patients^13^. Patients often die of obstructive jaundice, biliary tract infection, and hepatic failure, which are related to tumor progression.

Photodynamic therapy and local tumor ablation could improve bile drainage and keep biliary patency and have been proposed to prolong a patient’s survival^14,15^. A prospective cohort study compared systemic therapy to photodynamic therapy in 40 patients with successful biliary stenting. The photodynamic therapy group fared better with a median survival of 425 days, compared to 169 days for chemotherapy^15^.

IRE is a non-thermal ablation method that induces cell apoptosis by creating nanoscale-sized pores in cell membranes through the application of high-voltage, low-energy, direct-current pulses^16^. This method destroys only the cell membranes of tumor cells and does not damage the extracellular matrix components that maintain the continuity of sensitive structures, such as blood vessels, nerves, and bile ducts^5,17^. It is suitable for the treatment of tumors that are adjacent to or have infiltrated important structures, such as pancreatic cancer^18^.

In the past few years, we have used IRE for the treatment of hilar cholangiocarcinomas. We retrospectively reviewed the clinical data and outcomes of nine cases with hilar cholangiocarcinoma treated with IRE. This method appears to be a safe and feasible treatment because there were no serious adverse events or complications in these patients.

The transient elevation of CA19-9 on day 3 after the procedure might be related to extravasation of intracellular protein due to the perforation of cell membranes, but the steady normalization of CA19-9 thereafter indicated the effective targeting of tumor cells. All patients had neither biliary leakage nor vascular injury during the long-term follow-up. Our preliminary results revealed that there were no viable residual tumors in 88.9% of patients after IRE treatment, but obstruction of the bile ducts remained in the majority of patients, and PTCD drainage was needed to relieve jaundice. Only three of the nine (33.3%) patients were weaned off PTCD tubes. Nevertheless, all patients had a survival time that was longer than the expected average survival time reported in the current literature^19–22^.

We have proposed a new treatment strategy of using IRE for unresectable hilar cholangiocarcinoma, which addresses the dedicated local control of the primary tumor with a low risk non-thermal ablation procedure. Compared with other currently available palliative treatments, IRE has advantages of effective local tumor control, safety, fewer complications, and an absence of heat-sink effects^23,24^. Our preliminary results demonstrated high efficacy in local tumor control with an overall survival of 24.8 ± 6.84 months and disease progression-free survival of 18.5 ± 8.41 months, which exceeded the results of other standard palliative treatments that are currently available. To date, there has been a dearth of reports on the application of IRE in the treatment of hilar cholangiocarcinoma in the literature.

Martin et al. reported on 26 patients who had undergone IRE and demonstrated the method’s safety, with an acceptable complication rate of 11.5% (3/26). Following IRE, the median catheter-free time before requiring PTCD replacement was 305 days^25^. However, there have been no reports detailing the median or long-term survival of patients with hilar cholangiocarcinoma following IRE treatment. Our preliminary results demonstrate that IRE treatment without adjuvant chemotherapy likely prolongs patients’ overall and disease-free survival.

Our study has some limitations. We reported on a small number of cases with relatively short follow-up periods. Determining the long-term effectiveness requires larger case numbers with longer follow-up periods. However, our study still has clinical value for IRE-based treatment in the local control and progression of unresectable hilar cholangiocarcinoma.

Metastasis, tumor progression-related obstructive jaundice, and complications such as biliary infection due to external drainage tubes continue to play a crucial role in patient prognosis. Future work may consider whether IRE in combination with additional adjuvant chemotherapy and the replacement of PTCD tubes with internal biliary stents would be beneficial for patient prognosis. In conclusion, this study demonstrated that IRE appears to be a safe and feasible treatment for patients with unresectable hilar cholangiocarcinoma that sufficiently controls local tumor progression and may have potential benefits for patient survival.

## Data Availability

The data that support the findings of this study are available on request from the corresponding author.
